# Acupuncture and related therapies for treating stable angina pectoris: A protocol of an overview of systematic reviews and meta-analysis

**DOI:** 10.1097/MD.0000000000023701

**Published:** 2020-12-18

**Authors:** Haiju Sun, Xiaoyu Li, Jiali Lou, Yajun Zhang, Yongliang Jiang, Jianqiao Fang

**Affiliations:** a Department of Neurobiology and Acupuncture Research, The Third Clinical Medical College, Zhejiang Chinese Medical University, Key Laboratory of Acupuncture and Neurology of Zhejiang Province, Hangzhou City, Zhejiang Province; b The Third Affiliated Hospital of Zhejiang Chinese Medical University, Hangzhou, China.

**Keywords:** acupuncture, moxibustion, overview, protocol, stable angina pectoris

## Abstract

**Background:**

Stable angina pectoris (SAP) is a global health challenge. Multiple previous systematic reviews (SRs) have been conducted to assess the effectiveness of acupuncture and related therapies on SAP. We will carry out a comprehensive overview to map, synthesize, and assess the all the available evidence of acupuncture and related therapies on SAP.

**Methods:**

We will search 7 databases, including China National Knowledge Infrastructure (CNKI) and Chinese Biomedical Literature Database (CBM), WanFang Database, the Cochrane Library, PubMed, EMbase, MEDLINE. SRs and meta-analyses (MAs) of acupuncture and related therapies on SAP will be screened for eligibility. Systematic reviews, qualification evaluation, data extraction, methodological quality, and evidence quality evaluation will be conducted in pairs. The outcomes of interest include: frequency of angina attack, changes in nitroglycerin use, intensity of anginal pain, depression assessment, changes of the electrocardiogramme (ECG), anxiety assessment, results of the Six-Minute Walk Test (6-MWT), overall effectiveness, the Seattle Angina Questionnaire (SAQ), and adverse events. Where appropriate, the evidence will be synthesized based on the outcomes and patient subgroups.

**Results:**

This overview will be published in a peer-reviewed journal.

**Conclusion:**

This overview is expected to provide a reliable and valuable evidence of acupuncture for treating SAP.

**Ethics and communication:**

Given that this is an overview of published research, patient consent and ethical approval are not needed. The findings of this study will be disseminated through conference presentations and publication in peer-reviewed journals.

**PROSPERO registration number:**

CRD42020164466.

## 1 Introduction

### 1.1 Description of the condition

Stable angina pectoris (SAP), which is also named angina of effort, is a clinical syndrome of rapid and transient ischemia and hypoxia in the myocardium caused by the increase of myocardial load on the basis of severe coronary artery stenosis.^[1]^ The clinical syndrome of SAP involves pressure and discomfort in the left anterior chest region, severe pain, temporary hypoxic myocardial ischemia, and a burning sensation.^[2]^ And a growing number of clinical studies have shown SAP combined anxiety or depression impacted the quality of life seriously.^[3–5]^ Although the prognosis for patients with SAP varies, the annual mortality rate is up to 3.2%.^[6]^ According to the survey in America, SAP is widely prevalent, affecting about 10 million people and more than 500,000 cases were reported per year,^[7,8]^ and similar numbers were reported in Europe.^[9]^ In order to cope with the increased threat, diverse treatment options were used for SAP in clinical practice, such as pharmacological management, modification of lifestyle, risk factors elimination, and revascularization. And the economic cost of SAP is immense with an estimated 33 to 75 billion dollars annual spend.^[10]^ As SAP is a global health threat, multiple treatments are used to control the symptoms, improve the ischemia, and the prognosis, such as anti-ischemic drugs, revascularization, and control of risk factors.^[11]^ And acupuncture and related therapies, as alternative therapies in traditional Chinese medicine (TCM), have been proven to improve the pain symptoms, frequency of attacks and negative feelings in SAP patients.^[12]^

However, evidence of acupuncture and related therapies on SAP from existing systematic reviews (SRs) and meta-analyses (MAs) is inconsistent. While several SR/MAs suggested that acupuncture and related therapies could reduce the intensity and frequency of angina and nitroglycerin consumption,^[13–15]^ another SR showed acupuncture was effective on reducing the frequency of angina attacks and relief negative feelings in patients with SAP, but could not reduce the angina intensity and the nitroglycerin use.^[16]^ Moreover, the methodological quality of the previous studies is frequently suboptimal.

For these reasons, an overview will be carried out to synthesize and critically evaluate all clinical evidence on the comparative effectiveness of acupuncture and related therapies used either as adjuvant treatments or alone, compared with other conventional therapies of SAP by a MA approach.

### 1.2 Description of the objectives

The objectives of this overview are as follows:

1. Evaluate the quality of previous SR/MAs comprehensively, including methodological quality, evidence quality of SRs of acupuncture and related therapies on SAP, risk of bias among the included randomized clinical trials (RCTs), then point out what can be improved.
2. To summarize the existing evidence of acupuncture and related therapies on SAP for the effectiveness and to provide basis for acupuncture on SAP.

## 2 Methods

### 2.1 Study design and registration

The protocol has been registered in the international prospective register of SRs (PROSPERO, http://www.crd.york.ac.uk/PROSPERO, ID: CRD42020164466). The study will be conducted in accordance with the “preferred reporting items for overview of systematic reviews” (PRIO-harms).^[17]^

### 2.2 Eligibility criteria

#### 2.2.1 Types of studies

Previous SRs reported in English or Chinese will be considered for inclusion. Clinical reports, editor comments, reviews, correspondence, annual meeting abstracts, and narrative reviews will be excluded. To be eligible, SRs have to include meta-analysis results, and satisfy the participants, interventions, controls, and outcomes of interest criteria described below.

#### 2.2.2 Types of participants

Studies including patients diagnosed with SAP will be considered for inclusion. All eligible participants will be enrolled, regardless of age, race, and education background. Studies including following patients will be excluded: pregnant or lactating women; patients with concomitant severe diseases.

#### 2.2.3 Types of interventions

The intervention must be used at acupoints, trigger points or pain points. Manual acupuncture, electroacupuncture, moxibustion (any form of moxibustion), and acupressure will be included. Acupuncture plus moxibustion or cupping will also be included.

#### 2.2.4 Types of comparisons

Any SRs contained the following control treatments will be considered:

1. conventional pharmacological;
2. nonpharmacological treatments (including Chinese herbal medicine);
3. no treatment.

#### 2.2.5 Types of outcomes

Trials contained following outcomes will be considered:

1. frequency of angina attack;
2. changes in nitroglycerin use;
3. intensity of anginal pain;
4. depression assessment (scores of the Self-Rating Depression Scale (SDS));
5. changes of the electrocardiogramme (ECG);
6. anxiety assessment (scores of the Self-Rating Anxiety Scale (SAS));
7. results of the Six-Minute Walk Test (6-MWT);
8. overall effectiveness;
9. the Seattle Angina Questionnaire (SAQ);
10. adverse events.

### 2.3 Data collection

#### 2.3.1 Search methods for identification of reviews

Seven databases, including China National Knowledge Infrastructure (CNKI) and Chinese Biomedical Literature Database (CBM), WanFang Database, the Cochrane Library, PubMed, EMbase, MEDLINE, will be searched from inception to October 2020 for potentially eligible SRs. Only human researches will be included. Search terms are acupuncture, electroacupuncture, moxibustion, coronary disease, stable angina pectori, chronic coronary syndrome, systematic review, meta-analysis, meta-analysis. The search process is presented with a flow chart in Table 1.

**Table 1:** Search strategy for the PubMed database.

Query	Search term
#1	((“stable angina pectoris”[Mesh]) OR “chronic coronary syndrome”[Mesh]))
#2	(((“stable angina pectoris”[Title/Abstract]) OR “chronic coronary syndrome”[Title/Abstract])) OR “SAP”[Title/Abstract])
#3	#1 OR #2
#4	((((((“acupuncture”[Mesh]) OR “electroacupuncture”[Mesh]) OR “acupuncture therapy”[Mesh]) OR “acupuncture points”[Mesh]) OR “acupoint”[Mesh]) OR “moxibustion”[Mesh])
#5	((((((“acupuncture”[Title/Abstrac]) OR “electroacupuncture”[Title/Abstrac]) OR “acupuncture therapy”[Title/Abstrac]) OR “acupuncture points”[Title/Abstrac]) OR “acupoint”[Title/Abstrac]) OR “moxibustion”[Title/Abstrac])
#6	#4 OR #5
#7	((“meta-analysis as topic”[Mesh]) OR “systematic review”[Mesh])
#8	((“meta-analysis”[publication type]) OR “systematic review”[publication type])
#9	(((((“systematic review”[title/abstract]) OR “meta-analysis”[title/abstract]) OR “meta analysis”[title/abstract]) OR “meta-analyses”[title/abstract]) OR “meta analyses”[title/abstract])
#10	#7 OR #8 OR #9
#11	#3 AND #6 AND #10

#### 2.3.2 Studies selection

**Figure 1:**
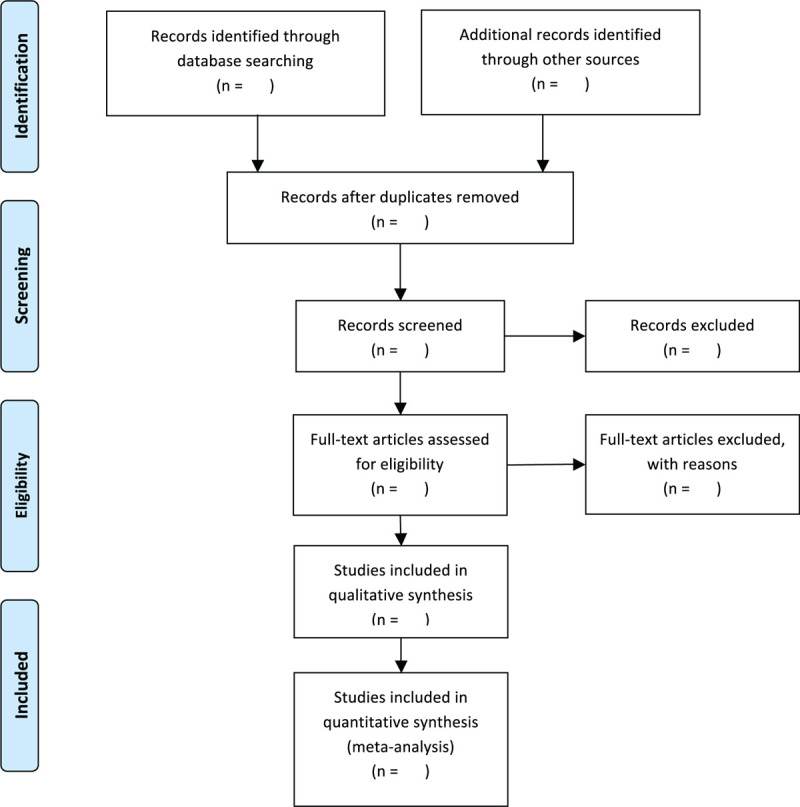
PRISMSA flow diagram of studies selection process.

Endnote X9 will be used to manage literatures and remove duplications. All titles and abstracts of the retrieved articles will be screened by 2 reviewers (Haiju Sun and Jiali Lou) independently, then the potential full texts will be evaluated, and determined eligibility. The full texts of each RCT included in the selected SRs will be get full tests. For duplicate citations, the latest version will be selected for the extraction. A third review author (Yongliang Jiang) will be invited to resolve disagreements. The flowchart of the selection process is presented on Figure 1.

#### 2.3.3 Data extraction and management

For each selected SRs, following data will be extracted independently and in duplicate by 2 reviewers (Xioayu Li and Yajun Zhang): titles, author(s), source of publication, publication year, authors name, country, sponsor, sample sizes, diagnostic criteria, inclusion and exclusion criteria, randomization methods, duration, interventions, blinding methods, results, dropout or withdrawal rates, outcome measures, adverse events and conclusion. A third reviewer (Yongliang Jiang) will be invited for judgment if disagreements are not resolved after discussion.

#### 2.3.4 Critical appraisal of included reviews

Two authors independently performed the following evaluation process (Haiju Sun and Xiaoyu Li). Any disagreements will be discussed at the meeting by all the authors until the consensus is reached. The rate of agreement between 2 assessors, which will be measured by Kappa statistic,^[18]^ will be calculated.

### 2.4 Data synthesis

The characteristics and findings of included SRs will be presented in a data extraction table and will be discussed in a narrative synthesis. These reviews will be synthesis, and the following pooled treatment effects will be provided: frequency of angina attack; changes in nitroglycerin use; intensity of anginal pain; 6-MWT; ECG; SAS; SDS; overall effectiveness; SAQ; adverse events. We will perform a subgroup analysis for each outcome, comparing acupuncture and related therapies vs drugs or vs sham control.

And if necessary, a meta-analysis will be summarized, using RevMan 5.3. For the quantitative synthesis, the treatment effect with the results for continuous outcome will be expressed as mean difference (MD) with 95% confidence interval (CI), and the relative risk (RR) with 95% CIs for dichotomous data. Heterogeneity will be assessed with the Chi-Squared test for each pooled analysis. Statistical significance will be considered to be a *P* value less than .10. The propitiation of total variations across the studies, caused by heterogeneity rather than change, will be described by the *I*^2^ value. A fixed-effect model will be used when *I*^2^ < 50%, otherwise, a random effect model will be used. If substantial heterogeneity was identified, sensitivity analyses or sensitivity analysis would be performed. If the source of heterogeneity remained undetermined, descriptive reports would be given.

### 2.5 Quality appraisal of the included reviews

#### 2.5.1 Methodological quality and reporting quality of included studies

The AMSTAR-2 tool will be used to critically appraise the systemic reviews. AMSTAR-2 is a measurement tool created to assess the methodological quality of systematic reviews. It consists of 16 items, whereby 7 of these are specific for critical areas (2, 4, 7, 9, 11, 13, 15). Based on the information contained in the SRs, the criteria will be rated as “yes”, “no”, “cannot report”, or “not applicable”. Each SR will be categorized into “high confidence” “moderate confidence”, “low confidence” or “critically low confidence”.^[19]^

Meanwhile, the report quality will be performed following the PRISMA checklist. PRISMA is a 27-item instrument, with each item rating on a 0–1 point scale. Every item can be answered with following possible choices: “yes”, “no” or “partly yes”, scored 1, 0, and 0.5, respectively. Score ranges are defined as follows: a score of below 11is low quality, 11–21 is moderate quality, and above 21 is high quality.^[20]^

Two reviewers (Jiali Lou and Yajun Zhang) will evaluate the methodological quality and the report quality independently. Any disagreements between the 2 reviewers will be resolved through discussion and consensus, and if required, a third author (Yongliang Jiang) will make an adjudication.

#### 2.5.2 Grading the quality of evidence

We will assess the quality of evidence using the GRADE System (Grading of Recommendations Assessment, Development and Evaluation, www.gradeworkinggroup.org) classify the evidence into insufficient-, low-, moderate-, or high-quality evidence.^[21]^ The estimated-risk and 95% CI of each outcome will be integrated into the table. The preliminary recommendations will be developed by 2 authors (Yajun Zhang and Jiali Lou).

#### 2.5.3 Dealing with missing data

If the data find to be unspecified, the author will be contacted by reviewers (Haiju Sun and Xiaoyu Li) to get the necessary information. The studies will be excluded if the missing data cannot be obtained, then we will assess the rest of the data and analysis the possible impact of missing information.

### 2.6 Protocol development and potential amendments

This overview of SR/MAs will be completed in strict accordance with this protocol. And if the protocol changed, the information and results will be shown in the final article.

## 3 Discussion

This research will be developed according to the standards and principles of evidence-based medicine. And this study will serve the purpose of clinicians on treating SAP, also on educating patients. At the same time, this research will contribute a lot to this field. First, as far as we are concerned, this is the first protocol to evaluate acupuncture and related therapies on SAP. Second, the results of this protocol will also be helpful to find out the interrelationship between the curative effect, time and frequency of acupuncture and related therapies and the site of acupuncture stimulation, improving the efficacy of acupuncture and related therapies on SAP. Third, acupuncturists will be able to formulate the optimal treatment plan for patients with SAP based on the results of this study.

Nevertheless, some limitations of this protocol have to be addressed. First, the application of acupuncture and related therapies has obvious regional limitation, most relevant researches are conducted in China. Second, the variance of acupuncture and related therapies processes and methods of operation among different regions should also be considered.

## Author contributions

**Conceptualization:** Haiju, Sun, Xiaoyu Li.

**Data curation:** Haiju, Sun.

**Formal analysis:** Haiju, Sun, Xiaoyu Li, Jiali Lou.

**Funding acquisition:** Jianqiao Fang.

**Investigation:** Jiali Lou, Yajun Zhang.

**Methodology:** Haiju, Sun, Xiaoyu Li, Jiali Lou, Yajun Zhang.

**Project administration:** Haiju, Sun, Xiaoyu Li, Jiali Lou, Yajun Zhang.

**Supervision:** Yongliang Jiang, Jianqiao Fang.

**Validation:** Jianqiao Fang.

**Visualization:** Haiju, Sun, Xiaoyu Li, Jiali Lou, Yajun Zhang.

**Writing – original draft:** Haiju, Sun.

**Writing – review & editing:** Yongliang Jiang, Jianqiao Fang.
